# IGCN: integrative graph convolution networks for patient level insights and biomarker discovery in multi-omics integration

**DOI:** 10.1093/bioinformatics/btaf313

**Published:** 2025-06-04

**Authors:** Cagri Ozdemir, Yashu Vashishath, Serdar Bozdag, Alzheimer’s Disease Neuroimaging Initiative, Cagri Ozdemir, Yashu Vashishath, Serdar Bozdag

**Affiliations:** Department of Computer Science and Engineering, University of North Texas, Denton, TX 76203, United States; BioDiscovery Institute, University of North Texas, Denton, TX 76203, United States; Center for Computational Life Sciences USA, University of North Texas, Denton, TX 76203, United States; Department of Computer Science and Engineering, University of North Texas, Denton, TX 76203, United States; BioDiscovery Institute, University of North Texas, Denton, TX 76203, United States; Center for Computational Life Sciences USA, University of North Texas, Denton, TX 76203, United States; Department of Computer Science and Engineering, University of North Texas, Denton, TX 76203, United States; BioDiscovery Institute, University of North Texas, Denton, TX 76203, United States; Center for Computational Life Sciences USA, University of North Texas, Denton, TX 76203, United States; Department of Mathematics, University of North Texas, Denton, TX 76203, United States

## Abstract

**Motivation:**

Developing computational tools for integrative analysis across multiple types of omics data has been of immense importance in cancer molecular biology and precision medicine research. While recent advancements have yielded integrative prediction solutions for multi-omics data, these methods lack a comprehensive and cohesive understanding of the rationale behind their specific predictions. To shed light on personalized medicine and unravel previously unknown characteristics within integrative analysis of multi-omics data, we introduce a novel integrative neural network approach for cancer molecular subtype and biomedical classification applications, named Integrative Graph Convolutional Networks (IGCN).

**Results:**

To demonstrate the superiority of IGCN, we compare its performance with other state-of-the-art approaches across different cancer subtype and biomedical classification tasks. Our experimental results show that our proposed model outperforms the state-of-the-art and baseline methods. IGCN identifies which types of omics data receive more emphasis for each patient when predicting a specific class. Additionally, IGCN has the capability to pinpoint significant biomarkers from a range of omics data types.

**Availability and implementation:**

The source code is available at https://github.com/bozdaglab/IGCN.

## 1 Introduction

Due to advancements in biotechnology, innovative omics technologies are constantly emerging, allowing researchers to access multi-layered information from genome-wide data. These multi-layered information can be obtained for the same set of samples, leading to generation of multi-omics datasets. Cancer molecular subtype prediction is a crucial area of research focused on classifying cancers into distinct subtypes based on their molecular characteristics. These subtypes can provide valuable insights into the biology of the cancer, predict patient outcomes, and guide personalized treatment strategies (Chaudhary *et al.* 2018, Poirion *et al.* 2018). Predicting cancer molecular subtypes through multi-omics integration may reveal complex interactions within biological systems and shed light on molecular mechanisms that contribute to cancer development and progression, which might be missed when examining a single type of omics data alone (Sharifi-Noghabi *et al.* 2019, Huang *et al.* 2019, Choi and Chae 2023, Khadirnaikar *et al.* 2023, Gong *et al.* 2023). Moreover, a multitude of approaches has shown that integration of multi-omics data can contribute to the precision medicine efforts in diseases such as Alzheimer’s disease (AD) (Shigemizu *et al.* 2020, Li *et al.* 2021, Abbas *et al.* 2022). While deep neural networks (NN)-based methods have been introduced as multi-omics integrative tools for cancer subtype prediction and diverse biomedical classification tasks (Chaudhary *et al.* 2018, Poirion *et al.* 2018, Sharifi-Noghabi *et al.* 2019, Huang *et al.* 2019, Choi and Chae 2023, Khadirnaikar *et al.* 2023, Gong *et al.* 2023), graph neural network (GNN)-based multi-omics integration approaches have shown promising results (Wang *et al.* 2021, Yin *et al.* 2022, Kesimoglu and Bozdag 2023, Xiao *et al.* 2023). Apart from NN-based tools, there is a set of methodologies that utilize matrix decomposition-based solutions for multi-modal biomedical data integration (Feng *et al.* 2018, Yang *et al.* 2023). Angle-based Joint and Individual Variation Explained (AJIVE) is a matrix decomposition method designed for effective multi-modal biomedical data integration (Feng *et al.* 2018). It separates shared and unique individual variation across datasets by analyzing subspaces and capturing how different data types vary across the same set of samples (e.g. patients). AJIVE also enables formal hypothesis testing to identify which features are statistically significant, as demonstrated in a real multi-omics data analysis (Yang *et al.* 2023). These matrix decomposition-based approaches ensure interpretable and identifiable integration, making it well-suited for understanding complex biological systems.

Multi-omics graph convolutional networks (MOGONET) has been introduced as a supervised multi-omics integration framework for a wide range of biomedical classification applications, which uses separate graph convolutional networks (GCN) for patient similarity networks based on mRNA expression, DNA methylation, and microRNA (miRNA) expression data types (Wang *et al.* 2021). MOGONET also utilizes View Correlation Discovery Network (VCDN) to explore the cross-omics correlations at the label space for effective multi-omics integration. Another computational tool named SUPREME, a subtype prediction methodology, integrates multiple types of omics data using GCN (Kesimoglu and Bozdag 2023). To obtain embeddings, SUPREME concatenates the features from all omics data types in the input space and utilizes them as node attributes in each GCN module to derive embeddings. Subsequently, SUPREME integrates these embeddings and conducts comprehensive evaluations of all possible combinations. Another recent approach, MOGAT (Tanvir *et al.* 2024), extends multi-omics integration by leveraging Graph Attention Networks (GAT). By incorporating attention-based message passing, MOGAT demonstrates improved performance in complex multi-omics tasks. Most recently, Trusted Multi-Omics integration framework based on hypergraph convolutional networks (called HyperTMO) has been developed (Wang *et al.* 2024). HyperTMO constructs hypergraph structures to represent the associations between samples in single-omics data. Following that, feature extraction is conducted utilizing a hypergraph convolutional network, while multi-omics integration occurs during the late stages of analysis.

Besides these GNN-based integrative tools applied for multi-omics datasets, several GNN-based approaches have been introduced as more general integrative computational tools for multi-modal datasets. Relational Graph Convolutional Networks (RGCN) (Schlichtkrull *et al.* 2018) provides relation-specific transformations, that is depending on the type and direction of an edge, for large-scale and multi-modal data. Heterogeneous Graph Attention Networks (HAN) (Wang *et al.* 2019) generates meta-path-based networks from a multi-modal network. The concept of meta-path can be applied to learn a sequence of relations defined between different objects in a multi-modal graph (Sun *et al.* 2011). After generating meta-paths, HAN takes node-level attention (for each node using its meta-path-based neighborhood) and association-level attention (for each meta-path) into consideration simultaneously. HAN employs a multi-layer perceptron (MLP) module to compute class probabilities rather than taking advantage of graph topologies, which could potentially limit the ability of the model to capture complementary information from graph topologies to make predictions. Apart from HAN, in the context of handling graph heterogeneity, Heterogeneous Graph Transformer (HGT) (Hu *et al.* 2020) has been developed to maintain representations that depend on node and edge types.

As outlined in Ferrari Dacrema *et al.* (2019), it has been argued that vanilla GCN and GAT methods could outperform existing integrative approaches after making some modifications to the networks. This underscores the necessity for more advanced computational methodologies in the integrative analysis of multi-omics data. In addition, the existing tools have some limitations. Omics data, by their nature, do not inherently exhibit a graph structure, thus a graph construction procedure is needed. However, constructing a graph from omics data could suffer from data noise due to many possible factors such as measurement inaccuracies, missing values, or inherent fluctuations within the dataset. These factors can adversely affect tasks like clustering, classification, or link prediction. Additionally, in the context of biomedical classification, different types of omics data have the potential to reveal unique characteristics at the label space. In other words, some omics types may demonstrate superior performance when predicting one disease label, while others might excel in predicting a different disease type. Therefore, directly fusing different types of omics data without considering the sample level importance of different omics networks may be liable to make wrong predictions and cause some level of performance degradation. Furthermore, multitudes of existing multi-omics integration tools do not explain how and why their models came to the prediction. In predictive modeling, a crucial trade-off arises: Do we merely desire the prediction, or are we interested in understanding the rationale behind it? (Doshi-Velez and Kim 2017). Since each of multi-omics type captures a different part of the underlying biology, understanding the ‘why’ can contribute to a deeper comprehension of the problem and advance the road toward precision medicine.

To address these limitations, we introduce a novel supervised integrative graph convolutional networks (IGCN) architecture that operates on multi-omics data structures. In IGCN, a multi-GCN module is initially employed to extract node embeddings from each network. A personalized attention module is then proposed to fuse the multiple node embeddings into a weighted form. Unlike previous multi-omics integration studies, the attention mechanism assigns different attention coefficients to each node/sample for each data modality to help identify which data modality receives more emphasis to predict a certain class type. This feature makes IGCN interpretable in terms of understanding the rationale behind the prediction at the sample level. Furthermore, IGCN has the capability to assign attention coefficients to features for each sample from a range of omics data types, which would facilitate identifying omics biomakers associated with phenotypes of interest. To the best of our knowledge, IGCN stands out as the first supervised integrative approach that provides patient level insights and biomarkers in multi-omics integration.

We presented our experimental results on four classification tasks: breast invasive carcinoma (BRCA) and glioblastoma (GBM) molecular subtype classification using The Cancer Genome Atlas dataset (Colaprico *et al.* 2016); Alzheimer’s Disease (AD) patients versus cognitively normal (CN) classification using The Religious Orders Study and Memory and Aging Project (ROSMAP) cohort (Hodes and Buckholtz 2016); and AD, Mild Cognitive Impairment (MCI), and CN classification task using Alzheimer’s Disease Neuroimaging Initiative (ADNI) dataset (Petersen *et al.* 2010). Our experimental results show that our proposed model outperforms the state-of-the-art and baseline methods. IGCN effectively identifies which omics data types are prioritized for each patient during class prediction. Additionally, IGCN has the capability to pinpoint significant biomarkers from a range of omics data types.

## 2 Materials and methods

### 2.1 Uncovering significant biomarkers during the prediction process

The feature ranking module in IGCN identifies noteworthy biomarkers across various omics datasets. Similar to the attention mechanism module given in Section 4.3, the feature ranking module also offers personalized insights regarding feature importance. The attention values for each feature can be computed as follows:


(1) $$r_{i}^{\left(j,k\right)}=\frac{\;exp(\sigma ((\sigma (x_{i}^{\left(j,k\right)}{\tilde{W}}_{i})){\hat{W}}_{i}+{\hat{b}}_{i}))}{\sum_{k=1}^{d_{i}}exp(\sigma ((\sigma (x_{i}^{\left(j,k\right)}{\tilde{W}}_{i})){\hat{W}}_{i}+{\hat{b}}_{i}))},$$

where $r_{i}^{\left(j,k\right)}\in R^{1}$ represents the attention value of the $k^{th}$ feature in the $i^{th}$ omics type corresponding to the $j^{th}$ sample. $x_{i}^{\left(j,k\right)}\in R^{1}$ is the $k^{th}$ feature in the $i^{th}$ omics type corresponding to the $j^{th}$ sample. ${\tilde{W}}_{i}\in R^{1\times \omega }$ and ${\hat{W}}_{i}\in R^{\omega \times 1}$ are the learnable weight matrices. ${\hat{b}}_{i}\in R^{1}$ is the bias parameter. σ is the activation function. As the input $x_{i}^{\left(j,k\right)}$ is a scalar, ${\tilde{W}}_{i}\in R^{1\times \omega }$ and ${\hat{W}}_{i}\in R^{\omega \times 1}$ were employed as an expansion and a compression units, respectively. $d_{i}$ is the feature size of the $i^{th}$ omics type and ω is the latent space dimension. Hence, we assign a feature rank for each feature used as an input. Moreover, since each sample was evaluated individually, the significance of each feature may differ for each patient.

### 2.2 Customizing GCN for omics-focused learning

IGCN employs GCN modules on graph networks to obtain the node embeddings. Each GCN module in IGCN can be defined as:


(2) $$H_{i}=\sigma (D_{i}^{-1/2}A_{i}D_{i}^{-1/2}(X_{i}⊗R_{i}\sqrt{d_{i}})W_{i}),$$

for i=1,2,…,p, where *p* is the total number of data modalities (omics types) and $X_{i}\in R^{m\times d_{i}}$ is the feature matrix (*m* is the number of nodes and $d_{i}$ is the feature size the $i^{th}$ omics type). $R_{i}\in R^{m\times d_{i}}$ is the feature ranking matrix computed in Equation (1). Specifically, we scale the attention values by $\sqrt{d_{i}}$. This follows the common practice in attention-based models to improve numerical stability and learning dynamics. The scaled attention values are then element-wise multiplied with the raw feature representations, ensuring that the model receives appropriately weighted feature inputs while preserving the relative importance captured by the attention mechanism. “⊗” denotes element-wise multiplication. $D_{i}$ and $W_{i}$ are the node degree and the learnable weight matrices, respectively. We used a single layer GCN to obtain the node embeddings ($H_{i}$) for each network layer, however, a multi-layer GCN can be considered as outlined in Kipf and Welling (2017).

In our work, following the approach in Wang *et al.* (2021), the original adjacency matrix $A_{i}\in R^{m\times m}$ was constructed by calculating cosine similarity of each node pair and filtering out edges with cosine similarity <ϵ. The adjacency matrix can be defined as:


(3) $$a_{i}^{\left(q,w\right)}=\mathrm{Ind}(s(x_{i}^{q},x_{i}^{w})),$$

where $x_{i}^{q}$ and $x_{i}^{w}$ are the node features of the node *q* and *w* (the $q^{th}$ and $w^{th}$ row vectors of $X_{i}$), respectively. $a_{i}^{\left(q,w\right)}$ is an element of the matrix $A_{i}$ corresponding to the $q^{th}$ row and $w^{th}$ column. $s(x_{i}^{q},x_{i}^{w})=\frac{\langle x_{i}^{q},x_{i}^{w}\rangle }{||x_{i}^{q}|{|}_{2}||x_{i}^{w}|{|}_{2}}$ is the cosine similarity. Ind(.) is an indicator function that maps the input to 1, if the input is greater than or equal to ϵ, and to 0 otherwise. As shown in Algorithm 1, the threshold ϵ can be determined based on a given parameter *k* as:


(4) $$k=\frac{1}{m}\sum_{q,w}Ind(s(x_{i}^{q},x_{i}^{w})).$$

Algorithm 1 Determine threshold ϵ based on pre-specified parameter *k*

1: **Input:** pre-specified parameter *k* and initialize ϵ=1

2: **Output:**  ϵ

3: **while true do**

4:  $\mathrm{counter}=\frac{1}{m}\sum_{q,w}Ind(s(x_{i}^{q},x_{i}^{w}))$

5:  **if**  counter>=k  **then**

6:   **break**

7:  **else**

8:   $\epsilon \leftarrow \epsilon -10^{-6}$

9:  **end if**

10: **end while**

Our approach for constructing the graph using the threshold ϵ determined by a pre-specified average degree *k* has several advantages over the traditional *k*-nearest neighbors (*k*NN)-based graph construction approach. Unlike *k*NN-based graphs, where each node is forced to have exactly *k* neighbors, our approach ensures that the average degree across all nodes matches the specified *k*. This allows for more flexibility in preserving the overall network structure. In *k*NN, nodes are always connected to the *k* most similar nodes, even if some of those connections are weak. However, our approach filters edges based on an absolute similarity threshold, ϵ, ensuring that only sufficiently strong connections are retained.

In our experiments, we empirically chose k=3 based on preliminary evaluations. Additionally, we conducted experiments with different *k* values to analyze the impact of varying similarity thresholds on model performance. The performance of IGCN, HyperTMO, MOGAT, SUPREME, MOGONET, RGCN, HGT, and HAN was evaluated on the TCGA-BRCA and ROSMAP datasets using similarity networks constructed with k=3,5,7, and 9. As shown in Fig. 3, available as supplementary data at *Bioinformatics* online, IGCN exhibited robustness to changes in *k* and consistently outperformed other integrative tools across different similarity thresholds.

When building a graph based on features and employing cosine similarity, it is frequent to come across data noise arising from diverse origins, such as measurement inaccuracies, missing values, or inherent fluctuations within the dataset. These inaccuracies can have negative impacts on node classification tasks. To alleviate these inaccuracies, the Normalized Temperature-scaled Cross Entropy loss (NT-Xent loss) (Sohn 2016) was utilized for each GCN module.


(5) $$pos_{q}=\sum_{j\in +}exp(s(x_{i}^{q},x_{i}^{j}))/\tau ,$$

(6) $$neg_{q}=\sum_{\ell \in -}exp(s(x_{i}^{q},x_{i}^{\ell }))/\tau .$$

In Equation (5), we calculate the cosine similarity between the anchor sample *q* and its positive pairs, scaled by the temperature parameter τ. Similarly, in Equation (6), we calculate the cosine similarity between the anchor sample *q* and its negative pairs, scaled by the temperature parameter. It is noteworthy that the anchor sample and its corresponding positive pairs belong to the same class, while the negative pairs were selected from classes different from that of the anchor sample. Thus, to learn representations that bring similar data points closer in the embedding space while pushing dissimilar data points farther apart, the NT-Xent loss for the $i^{th}$ GCN module can be defined as follows:


(7) $$L_{i_{NT-\mathrm{Xent}}}=\frac{1}{\upsilon }\sum_{q=1}^{\upsilon }log\left(\frac{pos_{q}}{pos_{q}+neg_{q}}\right).$$

where υ is the total number of samples in the training set.

### 2.3 Computing attention coefficients and predictions

After computing node embeddings using Equation (2), IGCN provides an attention mechanism to fuse multiple node embeddings into a weighted form by assigning attention coefficients to node embeddings. Inspired from (Veličković *et al.* 2017, Wang *et al.* 2019), attention coefficients can be determined as:


(8) $$c_{i}^{n}=\frac{\;exp(\sigma (h_{i}^{n}W_{a}+b))}{\sum_{j=1}^{p}exp(\sigma (h_{j}^{n}W_{a}+b))},$$

where $h_{i}^{n}\in R^{d}$ is the $n^{th}$ node embedding of the $i^{th}$ similarity network and *p* is the total number of data modalities. $W_{a}\in R^{d\times 1}$ and $b\in R^{1}$ are learnable weight and bias parameters, respectively. Attention coefficient $c_{i}^{n}\in R^{1}$ represents the importance of the $n^{th}$ node embedding of the $i^{th}$ network. Attention coefficients can be computed for all nodes of the similarity network and represented as a vector. Therefore, we can fuse the multiple node embeddings using element-wise multiplication, as follows:


(9) $$Z=\sum_{i=1}^{p}\vec{c_{i}}⊗H_{i},$$

where “⊗” denotes element-wise multiplication. $H_{i}$ is the node embedding matrix corresponding to $i^{th}$ similarity network and $\vec{c_{i}}$ is the attention coefficient vector for the nodes in the $i^{th}$ similarity network. It conveys that, although all embeddings are derived from a particular network, individual node embeddings may have distinct coefficient values. For example, consider the vector $\vec{c_{1}}$ as a column vector of size *m*:


$$\begin{array}{c} \vec{c_{1}}=\left[\begin{array}{c} c_{1}^{1}\\ c_{1}^{2}\\ \vdots \\ c_{1}^{m} \end{array}\right], \end{array}$$

and let $H_{1}$ be a matrix of size m×d:


$$\begin{array}{c} H_{1}=\left[\begin{array}{cccc} h_{1}^{\left(1,1\right)} & h_{1}^{\left(1,2\right)} & \ldots & h_{1}^{\left(1,d\right)}\\ h_{1}^{\left(2,1\right)} & h_{1}^{\left(2,2\right)} & \ldots & h_{1}^{\left(2,d\right)}\\ \vdots & \vdots & \ddots & \vdots \\ h_{1}^{\left(m,1\right)} & h_{1}^{\left(m,2\right)} & \ldots & h_{1}^{\left(m,d\right)} \end{array}\right]. \end{array}$$

We also note that $n^{th}$ row of $H_{1}$ can also be represented as a row vector $h_{1}^{n}$:


$$h_{1}^{n}=\left[\begin{array}{cccc} h_{1}^{\left(n,1\right)} & h_{1}^{\left(n,2\right)} & \ldots & h_{1}^{\left(n,d\right)} \end{array}\right].$$

The element-wise multiplication of each row of $\vec{c_{1}}$ by the corresponding row of $H_{1}$ can be represented as follows:


$$\begin{array}{c} \vec{c_{1}}⊗H_{1}=\left[\begin{array}{cccc} c_{1}^{1}\cdot h_{1}^{\left(1,1\right)} & c_{1}^{1}\cdot h_{1}^{\left(1,2\right)} & \ldots & c_{1}^{1}\cdot h_{1}^{\left(1,d\right)}\\ c_{1}^{2}\cdot h_{1}^{\left(2,1\right)} & c_{1}^{2}\cdot h_{1}^{\left(2,2\right)} & \ldots & c_{1}^{2}\cdot h_{1}^{\left(2,d\right)}\\ \vdots & \vdots & \ddots & \vdots \\ c_{1}^{m}\cdot h_{1}^{\left(m,1\right)} & c_{1}^{m}\cdot h_{1}^{\left(m,2\right)} & \ldots & c_{1}^{m}\cdot h_{1}^{\left(m,d\right)} \end{array}\right]. \end{array}$$

The weighted form of embeddings computed using Equation (9) is utilized on a neural network to obtain the node label predictions. Thus, it can be written as:


(10) $$\hat{Y}=\sigma (Z{\bar{W}}_{1}){\bar{W}}_{2},$$

where ${\bar{W}}_{1}$ and ${\bar{W}}_{2}$ are learnable weight matrices.

Besides the NT-Xent loss given in Equation (7), we also used the cross entropy loss as follows:


(11) $$L_{CE}=\sum_{j=1}^{v}-log\;\left(\frac{e^{\langle {\hat{y}}^{j},y^{j}\rangle }}{\sum_{k}e^{{\hat{y}}^{\left(j,k\right)}}}\right),$$

where ${\hat{y}}^{j}\in R^{d}$ is the $j^{th}$ row in $\hat{Y}$, which is the predicted label distribution of the $j^{th}$ training sample. ${\hat{y}}^{\left(j,k\right)}$ is the $k^{th}$ element in ${\hat{y}}^{j}$. $y^{j}$ is the one-hot encoded vector of the ground truth label of the $j^{th}$ training sample. $\langle {\hat{y}}^{j},y^{j}\rangle$ represents the inner product of the vector ${\hat{y}}^{j}$ and the vector $y^{j}$. To determine all learnable weights and biases, the total lost function can be written as:


(12) $$L_{\mathrm{IGCN}}=L_{CE}+\sum_{i=1}^{p}L_{i_{NT-\mathrm{Xent}}},$$

where *p* is the total number of data modalities.

Adam optimization (Kingma and Ba 2017) was used as the state-of-the-art for stochastic gradient descent algorithm and 0.5 dropout was added for each GCN layer. We evaluated all the methods on ten different randomly generated training, validation, and test splits. We selected 80% of the samples as the training set and 20% of the samples as the test set in a stratified fashion. We also used 25% of the training set as the validation set. Using the validation set, we performed hyperparameter tuning to determine the optimal hidden layer size, learning rate, and temperature value of NT-Xent loss function. Early stopping was used with the patience of 30 forced to have at least 200 epochs, which were determined empirically.

## 3 Results

### 3.1 Overview of IGCN architecture

**Figure 1.**
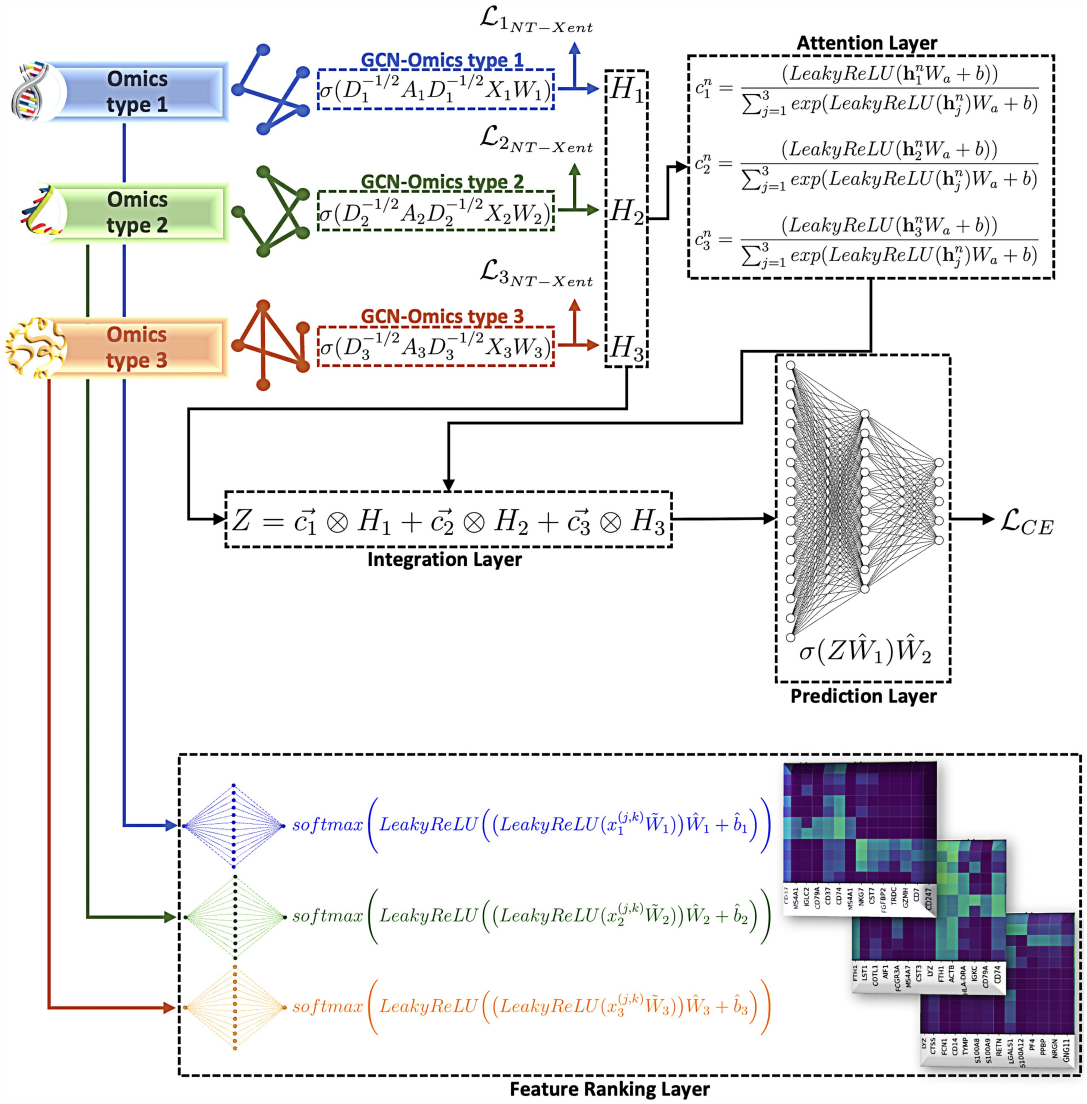
Overview of IGCN architecture on three similarity networks. IGCN employs an integration module to fuse the node embeddings. Simultaneously, IGCN assigns attention coefficients to features for individual samples across diverse omics data types.

IGCN integrates multi-omics data and reveals patient level insights regarding both the key omics types and features for biomedical classification tasks. The overview of IGCN architecture is illustrated in Fig. 1. We first construct graphs for each omics type [Equations (3) and (4)]. Subsequently, IGCN utilizes GCN modules on graphs to learn the node embeddings. As depicted in Algorithm 1, the hyperparameter ϵ determines a threshold for correlation in graph construction. In graph construction, it is common to encounter data noise from various sources, such as measurement inaccuracies, missing values, or inherent dataset fluctuations. To alleviate this noise, the NT-Xent loss [Equation (7)] is utilized for each GCN module. Following this, we introduce a personalized attention module to merge the multiple node embeddings into a weighted representation [Equations (8) and (9)]. Differing from prior research, this attention mechanism assigns distinct attention coefficients to each patient, facilitating the identification which data modality is more influential in predicting a particular class type. The integrated embeddings are fed into an MLP for prediction, referred to as the prediction layer in Fig. 1. Similar to the attention mechanism module, the feature ranking module also offers personalized insights regarding feature importance [Equation (1). It assigns attention coefficients to features, computed specifically for each patient. These coefficients are then used to weight the node features in the GCN layers.

To our knowledge, IGCN is the first supervised integrative approach that offers patient-level insights and biomarkers in multi-omics integration, making it a significant advancement in the field.

### 3.2 Biomedical classification tasks

To perform BRCA subtype classification task and prediction between AD and CN cases, we used TCGA-BRCA and ROSMAP datasets, respectively. For these datasets, preprocessed mRNA expression, DNA methylation, and miRNA expression data were collected from http://github.com/txWang/MOGONET. As outlined in Wang *et al.* (2021), to filter out features, different variance filtering thresholds were applied for different types of omics data as each omics type has different ranges. For mRNA expression and DNA methylation data types, to filter out low-variance features, thresholds of 0.1 and 0.001 were used, respectively. For miRNA expression data, features with no variation (i.e., variance equal to zero) were filtered out. PAM50 labels of the tumor samples were obtained as ground truth class labels (Parker *et al.* 2009) for BRCA subtype classification. Specifically, we have five different class labels: Basal-like, HER2-Enriched, Luminal A, Luminal B, and Normal-like.

For GBM molecular subtype classification task, we acquired mRNA expression and miRNA expression data from the Broad Institute’s Firehose data portal (Deng *et al.* 2017) (available at http://firebrowse.org/). DNA methylation data was not used because most of the samples available for other data modalities were not available for DNA methylation, leading to a small sample size. To preprocess mRNA expression data, we filtered mRNAs with ≤0.1 variance. Similarly, for miRNA expression data, features with no variation (i.e. variance equal to zero) were filtered out. We utilized the ground truth labels provided in Verhaak *et al.* (2010), which identify four molecular subtypes based on gene expression, namely Proneural, Neural, Mesenchymal, and Classical.

We also collected diverse omics types, namely single nucleotide polymorphisms (SNPs), lipidomics, and bileomics, from ADNI dataset (Petersen *et al.* 2010) and conducted a classification task to predict AD, MCI, and CN individuals. We processed the genetic data by merging SNPs information of all patients into a single set of files using PLINK software package (Weeks 2010). SNPs that have missing call rate ≤95%, had minor allele frequency (MAF) ≤5%, or with Hardy–Weinberg equilibrium test *P*-value $>10^{-6}$ were removed. Samples with call rate <95% were removed. To identify SNPs associated with the disease labels (i.e. CN, MCI, and AD), we performed a Genome-Wide Association Study (GWAS). For each SNP, we computed the negative logarithm of its *P-*value in GWAS. We identified 156 variants that exhibited statistically significant association with the disease labels. For lipidomics, the features that had a high correlation (≥0.9) were eliminated, reducing the number of the features from 781 to 637. For bileomics, we performed several quality control steps to ensure accuracy and consistency. These included adjusting for batch effects, filling in missing values through imputation, and applying a negative log base 2 transformation to normalize the data. Since the dataset originally had only 24 features, we did not perform feature reduction, as reducing the feature count further could have limited the available information. A detailed description of the datasets can be found in Table 1, available as supplementary data at *Bioinformatics* online.

**Table 1.** Classification results on TCGA-BRCA, TCGA-GBM, ROSMAP, and ADNI datasets.^a^

Dataset	Method	Accuracy	Weighted F1	Macro F1	MCC
TCGA-BRCA	MLP	0.761±0.012	0.752±0.015	0.704±0.022	0.648±0.020
	SVM	0.774±0.022	0.771±0.022	0.736±0.027	0.668±0.033
	RF	0.714±0.020	0.690±0.023	0.594±0.036	0.565±0.032
	GCN	0.787±0.012	0.782±0.016	0.743±0.024	0.685±0.020
	GAT	0.789±0.015	0.785±0.017	0.747±0.022	0.688±0.024
	HAN	0.781±0.025	0.772±0.036	0.714±0.069	0.675±0.039
	HGT	0.795±0.028	0.789±0.030	0.739±0.044	0.697±0.042
	RGCN	0.825±0.017	0.824±0.020	0.791±0.032	0.744±0.027
	MOGONET	0.813±0.013	0.813±0.014	0.765±0.027	0.727±0.019
	SUPREME	0.821±0.020	0.822±0.022	0.783±0.032	0.742±0.031
	MOGAT	0.837±0.018	0.839±0.017	0.806±0.016	0.763±0.026
	HyperTMO	0.838±0.015	0.841±0.016	0.813±0.025	0.768±0.022
	**IGCN**	**0.874 ± 0.011**	**0.878 ± 0.010**	**0.852 ± 0.017**	**0.821 ± 0.014**
TCGA-GBM	MLP	0.793±0.048	0.791±0.050	0.783±0.050	0.725±0.063
	SVM	0.779±0.057	0.777±0.056	0.767±0.058	0.706±0.075
	RF	0.818±0.049	0.811±0.055	0.801±0.059	0.761±0.063
	GCN	0.880±0.017	0.879±0.017	0.873±0.018	0.839±0.023
	GAT	0.861±0.018	0.859±0.019	0.852±0.021	0.814±0.025
	HAN	0.858±0.038	0.856±0.041	0.850±0.044	0.809±0.051
	HGT	0.840±0.033	0.840±0.034	0.839±0.036	0.788±0.045
	RGCN	0.851±0.043	0.849±0.045	0.845±0.049	0.801±0.057
	MOGONET	0.854±0.019	0.854±0.020	0.851±0.023	0.805±0.026
	SUPREME	0.818±0.030	0.816±0.034	0.808±0.041	0.756±0.041
	MOGAT	0.817±0.037	0.815±0.039	0.808±0.043	0.755±0.049
	HyperTMO	0.837±0.026	0.836±0.026	0.832±0.029	0.781±0.034
	**IGCN**	**0.903 ± 0.014**	**0.902 ± 0.014**	**0.898 ± 0.013**	**0.870 ± 0.019**
ROSMAP	MLP	0.657±0.056	0.650±0.059	0.650±0.059	0.335±0.111
	SVM	0.645±0.063	0.623±0.079	0.621±0.081	0.308±0.131
	RF	0.692±0.066	0.691±0.065	0.691±0.066	0.387±0.136
	GCN	0.701±0.042	0.700±0.042	0.699±0.042	0.405±0.085
	GAT	0.670±0.032	0.669±0.033	0.669±0.033	0.347±0.064
	HAN	0.775±0.025	0.775±0.025	0.774±0.025	0.550±0.052
	HGT	0.758±0.022	0.756±0.022	0.756±0.022	0.527±0.041
	RGCN	0.744±0.024	0.741±0.024	0.740±0.024	0.503±0.049
	MOGONET	0.782±0.019	0.781±0.019	0.781±0.019	0.571±0.037
	SUPREME	0.782±0.028	0.781±0.028	0.781±0.028	0.575±0.056
	MOGAT	0.758±0.030	0.757±0.031	0.757±0.031	0.521±0.058
	HyperTMO	0.796±0.035	0.795±0.035	0.795±0.035	0.596±0.071
	**IGCN**	**0.824 ± 0.034**	**0.823 ± 0.034**	**0.823 ± 0.034**	**0.659 ± 0.069**
ADNI	MLP	0.774±0.028	0.770±0.029	0.770±0.029	0.660±0.041
	SVM	0.766±0.045	0.763±0.048	0.762±0.048	0.651±0.065
	RF	0.791±0.047	0.788±0.048	0.788±0.049	0.689±0.071
	GCN	0.783±0.037	0.782±0.038	0.785±0.037	0.677±0.054
	GAT	0.760±0.036	0.759±0.035	0.763±0.034	0.642±0.055
	HAN	0.799±0.032	0.800±0.031	0.802±0.029	0.702±0.047
	HGT	0.758±0.039	0.757±0.041	0.762±0.041	0.642±0.056
	RGCN	0.807±0.034	0.806±0.034	0.808±0.033	0.713±0.050
	MOGONET	0.733±0.033	0.732±0.034	0.735±0.035	0.601±0.049
	SUPREME	0.803±0.036	0.803±0.038	0.806±0.037	0.709±0.052
	MOGAT	0.798±0.038	0.797±0.038	0.799±0.038	0.700±0.056
	HyperTMO	0.794±0.027	0.793±0.025	0.796±0.025	0.695±0.042
	**IGCN**	**0.840 ± 0.026**	**0.840 ± 0.026**	**0.842 ± 0.026**	**0.762 ± 0.039**

a The reported values represent the averages along with standard deviations, based on ten runs, for four performance measures, namely: accuracy, macro F1, weighted F1, and Matthew’s correlation coefficient (MCC). The best values for each dataset are shown in bold. The underline is used to signify the second-best performance.

### 3.3 IGCN demonstrated superior performance in various cancer molecular subtype prediction and biomedical classification tasks

We compared the performance of IGCN with the state-of-the-art methods (i.e. GCN, GAT, HAN, HGT, RGCN, MOGONET, MOGAT, SUPREME, and HyperTMO) as well as baseline methods, namely MLP and Random Forest (RF), and Support Vector Machine (SVM). We evaluated their performance based on four metrics: accuracy, macro F1 score, weighted F1 score, and Matthew’s correlation coefficient (MCC). We evaluated all the methods on ten different randomly generated training, validation, and test splits. We selected 80% of the samples as the training set and 20% of the samples as the test set in a stratified fashion. We also used 25% of the training set as the validation set to tune the hyperparameters (e.g. hidden layer size and learning rate) and perform early stopping.

The quantitative results of our comparative experiments, presented in Table 1, show that IGCN achieved the best performance across all metrics and datasets. Since GCN, GAT, MLP, SVM, and RF are not integrative tools, we evaluated each data modality separately and presented the best results for each method. These results highlight the importance of multi-omics integration because even the top performance achieved is still not superior to that of IGCN.

In Ferrari Dacrema *et al.* (2019), it was shown that given proper inputs, simple homogeneous GNN-based integration approaches, such as GCN and GAT, may surpass the performance of all existing integrative tools across various scenarios. Similarly, our experimental results show that GCN delivers the second-best performance on GBM dataset, outperforming integrative methods such as HAN, HGT, RGCN, MOGONET, MOGAT, SUPREME, and HyperTMO. Furthermore, both GCN and GAT outperformed both MOGONET and HGT on ADNI dataset. However, IGCN demonstrated the best classification performance across all dataset, representing a more sophisticated and advanced integrative tool.

**Figure 2.**
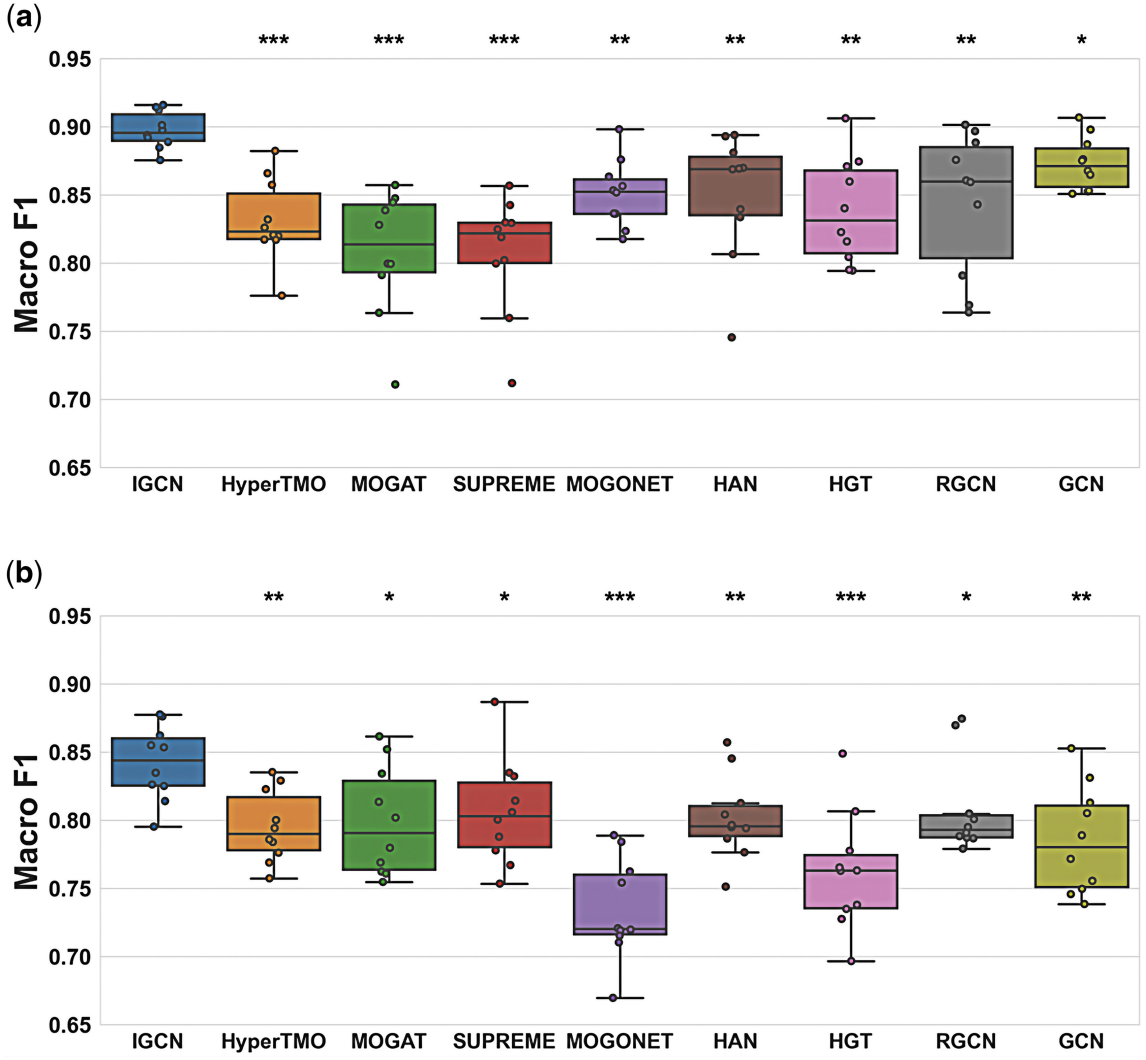
The boxplots show the distribution of macro F1 scores of ten different runs on (a) TCGA-GBM and (b) ADNI datasets for all methods. The means and standard deviations of these runs are shown in Table 1. Wilcoxon rank-sum test *P*-values were computed between IGCN and other methods to compare the distribution of box plots, representing *P*-value < 0.001 by ***, else if < 0.01 by **, and else if < 0.05 by *.

In Fig. 2a and b, the boxplots show the distribution of macro F1 scores of ten runs on GBM and ADNI datasets. Table 1 presents the means and standard deviations of these runs. We also calculated Wilcoxon rank-sum test p-values to compare the distribution of the box plots between IGCN and other methods. For TCGA-GBM, ADNI, and TCGA-BRCA datasets, as shown in Fig. 2 and Fig. 1a, available as supplementary data at *Bioinformatics* online, IGCN outperforms all other methods significantly (*P*-value < 0.05). For ROSMAP dataset, as shown in Fig. 1b, available as supplementary data at *Bioinformatics* online, we observed statistically significant difference with all the methods (*P*-value < 0.05) except for HyperTMO (*P*-value > 0.05).

### 3.4 Attention-driven interpretability in IGCN

**Figure 3.**
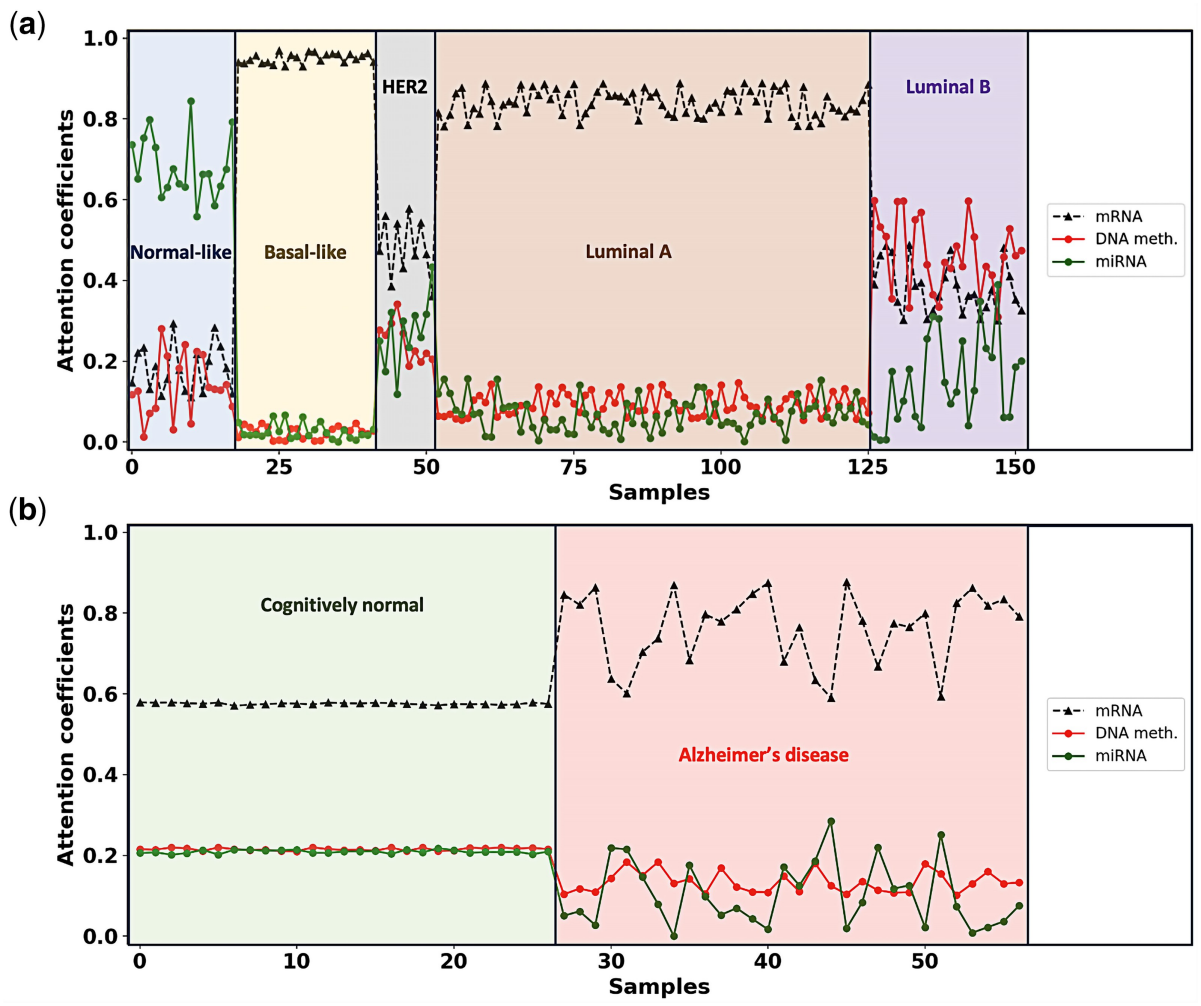
Attention coefficients of correctly predicted test samples of **(a)** TCGA-BRCA and **(b)** ROSMAP datasets. IGCN provides an attention mechanism, which computes a specific attention coefficient for each node embedding. This specialty might allow us to investigate which feature is most informative for each sample in different node type prediction.

The personalized attention mechanism in IGCN is proposed to integrate multi-omics data embeddings into a weighted form. As this module assigns specific attention coefficients to each sample, we can observe unique characteristic pattern of each omics type at the label space. The significance of different embeddings derived from various types of omics data networks varies from sample to sample, depending on the cancer molecular type or specific diagnosis group. Figure 3a and b show the attention coefficients computed for all correctly predicted test samples in TCGA-BRCA and ROSMAP datasets, respectively. For TCGA-BRCA dataset, mRNA expression data had the main contribution toward the prediction of Basal-like, HER2-enriched, and Luminal A breast cancer subtypes, which is expected as PAM50 subtypes were defined based on mRNA expression data. Interestingly, however, miRNA expression data made the main contribution to predicting Normal-like breast cancer subtype. It is also notable that the attention level of DNA methylation data is slightly higher compared to the attention level of mRNA expression data in Luminal B samples. Concerning ROSMAP dataset, mRNA expression plays a primary role in both CN and AD samples. However, the attention level given to mRNA expression data is slightly higher for AD samples compared to CN samples. The attention level of each omics type varies across different samples. It is apparent that various types of omics data are integrated according to a patient-specific golden ratio. This feature makes IGCN interpretable in terms of understanding the rationale behind the prediction at the sample level. Moreover, it has the potential to pave the way for a new research direction in analyzing different omics data types on different cancer subtype samples.

In addition to the integration layer, the feature ranking layer shown in Fig. 1 assigns attention coefficients to input features across all omics types. The feature ranking layer is unique such that it assigns attention values customized to each individual sample. This indicates that rather than employing a global attention mechanism that treats all samples uniformly, the module personalizes the attention scores for each individual sample. As illustrated in Fig. 2, available as supplementary data at *Bioinformatics* online, the attention values for miRNA features in ROSMAP dataset exhibit variability across miRNAs. We enforce the normalization of attention values [given in Equation (1)], ensuring that their cumulative sum amounts to 1 for each sample. Considering the attention scores across the features, it can be inferred that the features with relatively higher attention scores hold greater significance compared to others. For TCGA-BRCA and ROSMAP datasets, we ranked omics features based on their average attention values within each subtype or disease type and reported the top ten biomarkers in Tables 2 and 3, respectively. IGCN identified unique as well as common biomarkers for each subtype or disease type, highlighting their distinct roles in the prediction. Recent studies have provided evidence supporting the involvement of these biomarkers in the pathogenesis of breast cancer and AD (Esteras *et al.* 2012, Dai *et al.* 2015, Nami and Wang 2018, Garcia-Escolano *et al.* 2021, Martin *et al.* 2021, Xie *et al.* 2022, Sharma *et al.* 2023, Shin *et al.* 2023, S and Sundararajan 2025).

**Table 2.** The highest-ranked 10 omics biomarkers identified through IGCN in TCGA-BRCA dataset for each subtype are presented in rank order from left to right.^a^

Subtype	Biomarkers
Normal-like	**mRNA: ENO1**, **TMBIM6**, IGFBP4, SPARCL1, XBP1, **TUBB**, SFRP1, MYH11, CYBRD1, SYNM
	**miRNA: hsa-miR-148a**, **hsa-miR-22**, **hsa-miR-143**, **hsa-miR-21**, hsa-miR-30a, **hsa-miR-10b**, hsa-let-7b, **hsa-miR-99b**, hsa-miR-10a, hsa-let-7a-2
	**DNA methylation: ID1**, TMEFF1, GAS1, **C2orf52**, DOK5, PSAT1, LOC80054, POU4F1, FOXA1, CEBPA
Basal-like	**mRNA: ENO1**, **TUBB**, HDGF, **TMBIM6**, PDIA6, GABRP, YBX1, SFRP1, KRT6B, LDHB
	**miRNA: hsa-miR-22**, **hsa-miR-21**, **hsa-miR-148a**, hsa-miR-30a, **hsa-miR-99b**, **hsa-miR-143**, **hsa-miR-10b**, hsa-miR-182, hsa-miR-103–1, hsa-miR-203
	**DNA methylation:** FOXC1, **ID1**, TMEFF1, **C2orf52**, SFT2D2, FOXD1, ABHD3, GAS1, SCGB3A1, DOK5
HER2	**mRNA:** XBP1, **TUBB**, **TMBIM6**, **ENO1**, ERGIC1, HDGF, MAGED2, RHOB, YBX1, GSTP1
	**miRNA:** hsa-miR-182, **hsa-miR-10b**, **hsa-miR-21**, **hsa-miR-143**, **hsa-miR-22**, **hsa-miR-148a**, **hsa-miR-99b**, hsa-miR-183, hsa-let-7b, hsa-miR-10a
	**DNA methylation:** PSAT1, **ID1**, FOXA1, ABHD3, **C2orf52**, FOXD1, TMEFF1, GAS1, BCL2L1, LOC285548
Luminal A	**mRNA: TMBIM6**, XBP1, IGFBP4, **TUBB**, MAGED2, SLC39A6, RHOB, **ENO1**, GATA3, BTG2
	**miRNA: hsa-miR-21**, **hsa-miR-22**, **hsa-miR-10b**, hsa-miR-30a, hsa-miR-182, **hsa-miR-143**, hsa-let-7b, **hsa-miR-99b**, **hsa-miR-148a**, hsa-miR-10a
	**DNA methylation:** FOXA1, **ID1**, BCL2L1, ABHD3, SIAH2, **C2orf52**, FAM63A, BHLHE40, SOS2, PPP4R1
Luminal B	**mRNA:** XBP1, **TMBIM6**, **TUBB**, SLC39A6, MAGED2, ESR1, **ENO1**, IGFBP4, GATA3, CA12
	**miRNA: hsa-miR-10b**, **hsa-miR-21**, **hsa-miR-22**, **hsa-miR-99b**, **hsa-miR-143**, **hsa-miR-148a**, hsa-miR-30a, hsa-miR-182, hsa-let-7b, hsa-miR-103–1
	**DNA methylation:** FOXA1, **ID1**, SIAH2, BCL2L1, **C2orf52**, BHLHE40, FAM63A, TMEFF1, ABHD3, SOS2

a Unique mRNA, miRNA, and DNA methylation biomarkers are underlined. Biomarkers that are common across all subtypes are boldfaced.

**Table 3.** The highest-ranked 10 omics biomarkers identified through IGCN in ROSMAP dataset for each disease type are presented in rank order from left to right.^a^

Subtype	Biomarkers
Alzheimer’s disease	**mRNA: CDKN1A**, **QDPR**, **CCND1**, **CAV1**, **BCL2**, **BAX**, **VEGFA**, **PPDPF**, **PTEN**, SLC22A3
	**miRNA: hsa-miR-26a**, **hsa-miR-29a**, **hsa-miR-126**, **hsa-let-7g**, **hsa-miR-125b**, **hsa-miR-9**, **hsa-miR-16**, **hsa-let-7b**, **hsa-miR-99a**, **hsa-miR-30b**
	**DNA methylation: C20orf152**, **ABCB5**, **ATP2C2**, **TMEM61**, **HAO2**, **MYPN**, **LRRC39**, **VHL**, **STOM**, OLFML3
Cognitively normal	**mRNA: QDPR**, **PPDPF**, **CAV1**, **CDKN1A**, **CCND1**, **PTEN**, **VEGFA**, GAPDH, **BAX**, **BCL2**
	**miRNA: hsa-let-7b**, **hsa-miR-26a**, **hsa-miR-29a**, **hsa-let-7g**, **hsa-miR-125b**, **hsa-miR-126**, **hsa-miR-9**, **hsa-miR-30b**, **hsa-miR-99a**, **hsa-miR-16**
	**DNA methylation: TMEM61**, **ABCB5**, **ATP2C2**, **C20orf152**, **MYPN**, **LRRC39**, **HAO2**, **VHL**, C19orf54, **STOM**

a Unique mRNA, miRNA, and DNA methylation biomarkers are underlined. Biomarkers that are common across all subtypes are boldfaced.

For TCGA-BRCA dataset, the genes ENO1, TMBIM6, TUBB, ID1, and C2orf52 have been identified as key biomarkers shared across all molecular subtypes. Specifically, ENO1, TMBIM6, and TUBB were selected in mRNA expression, while ID1 and C2orf52 were identified through DNA methylation. All these genes have been implicated in various breast cancer studies (Nami and Wang 2018, Garcia-Escolano *et al.* 2021, Sharma *et al.* 2023, Shin *et al.* 2023). ID1 has been identified as the most important gene for the Normal-like subtype and the second most important gene across all other breast cancer subtypes (Table 2). As outlined in Garcia-Escolano *et al.* (2021), ID1 has been shown to be associated with tumor aggressiveness and poor prognosis across different molecular subtypes of breast cancer. Additionally, ID1 expression has been linked to increased tumor invasiveness and metastatic potential, highlighting its critical role in cancer progression (Garcia-Escolano *et al.* 2021). These findings support our results, reinforcing the significance of ID1 as a biomarker across breast cancer molecular subtypes. Furthermore, FOXA1 and XBP1 genes have been identified as key biomarkers across all breast cancer subtypes except Basal-like subtype. FOXA1 gene has been shown to play a critical role in the subtyping and carcinogenesis of breast tumors (Dai *et al.* 2015). FOXA1 is also one of the PAM50 genes, which were utilized to create PAM50 subtypes (Parker *et al.* 2009). In addition to FOXA1, we also observed that three additional PAM50 genes, namely FOXC1, SFRP1, and SLC39A6, have been identified as top biomarkers in TCGA-BRCA dataset (Table 2). FOXC1 has been recognized as the most important biomarker and appears only in Basal-like patients. SFRP1 has been identified in both the Normal-like and Basal-like patients, while SLC39A6 appears in both the Luminal A and Luminal B patients.

Additionally, we reported biomarkers that are unique to each subtype. Specifically, SPARCL1, MYH11, CYBRD1, SYNM, LOC80054, POU4F1, and CEBPA were identified for Normal-like subtype; PDIA6, GABRP, KRT6B, LDHB, FOXC1, SFT2D2, and SCGB3A1 for Basal-like; ERGIC1, GSTP1, and LOC285548 for HER2; BTG2 and PPP4R1 for Luminal A; and ESR1 and CA12 for Luminal B. To check if these subtype-specific biomarkers have unique expression/DNA methylation in their corresponding subtype, we compared the distribution of expression/DNA methylation of these genes to the distribution in the other subtypes (Figs 4–8, available as supplementary data at *Bioinformatics* online). We observed that in most cases, the biomarker was upregulated/hypermethylated or downregulated/hypomethylated with respect to other subtypes. These results support IGCN findings on their role in distinguishing molecular subtypes.

The miRNAs hsa-miR-22, hsa-miR-143, hsa-miR-21, hsa-miR-10b, and hsa-miR-99b have also been recognized as important biomarkers that are present across all breast cancer molecular subtypes. hsa-miR-22 plays a dual role in breast cancer by both promoting and suppressing cell proliferation, apoptosis, and cell cycle progression (Fan *et al.* 2021). As detailed in Kumar *et al.* (2013) and Niedźwiecki *et al.* (2019), hsa-miR-21 and hsa-miR-10b have been detected as one of the most significantly upregulated miRNAs in breast cancer samples. Notably, hsa-miR-21 is strongly associated with advanced clinical stages, lymph node metastasis, and poorer survival outcomes in breast cancer patients, underscoring its potential as a key prognostic marker in breast cancer management (Yan *et al.* 2008). Additionally, hsa-miR-143 has been found to suppress cell proliferation and invasion in breast cancer by targeting ERBB3 (Yan *et al.* 2014).

For ROSMAP dataset, CDKN1A, QDPR, CCND1, CAV1, BCL2, BAX, VEGFA, PPDPF, and PTEN genes have been identified as important biomarkers across both AD and CN groups. In AD, beta-amyloid (Aβ) peptides can aggregate and accumulate, forming insoluble plaques in the brain. These plaques are a hallmark pathological feature of AD and are believed to contribute to the progressive neurodegeneration and cognitive decline characteristic of the disease. CDKN1A and VEGFA have been implicated in processes related to Aβ accumulation in AD (Esteras *et al.* 2012, Martin *et al.* 2021). QDPR, CCND1, CAV1, BCL2, BAX, PPDPF, PTEN, C20orf152, ABCB5, ATP2C2, TMEM61, HAO2, MYPN, LRRC39, VHL, and STOM genes have potential as biomarkers for AD diagnosis and prediction of conversion from CN to AD. Their expression profiles may provide insights into underlying pathological processes such as Aβ accumulation, apoptosis, vascular dysfunction, and neuronal cell cycle dysregulation (Kitamura *et al.* 1998, Gaudreault *et al.* 2004, Sonoda *et al.* 2010, Mathys *et al.* 2019). However, further research and validation in clinical cohorts are needed to confirm their utility in AD prediction.

hsa-let-7b, hsa-miR-26a, hsa-miR-29a, hsa-let-7g, hsa-miR-125b, hsa-miR-126, hsa-miR-9, hsa-miR-30b, hsa-miR-99a, hsa-miR-16 have been identified as key miRNA biomarkers common to all molecular subtypes. Recently, hsa-miR-29a and hsa-miR-26a have been identified as a key regulatory molecule linking AD, highlighting its potential as a biomarker for AD (Xie *et al.* 2022, Sundararajan 2025). The study demonstrated that hsa-miR-125b is differentially expressed in the serum-derived exosomes of AD patients compared with healthy subjects (Duan *et al.* 2024). hsa-miR-126 has been introduced as an angiogenic miRNA that regulates vascular function and plays a crucial role in cognitive and glymphatic function in mice with vascular dementia (VaD). Its reduction in endothelial cells leads to cognitive impairment, decreased cerebral blood flow, and increased inflammation (Yu *et al.* 2019). VaD is primarily caused by reduced blood flow to the brain due to multiple small strokes or microinfarctions, whereas AD is driven by Aβ plaques and tau tangles. However, hsa-miR-126’s role in regulating vascular function and glymphatic clearance could have implications for both VaD and AD, as vascular dysfunction is also a contributing factor in AD (Fisher *et al.* 2022). Moreover, as outlined in Poursaei *et al.* (2022), the expression level of hsa-let-7g was significantly higher in AD patients compared to CN individuals.

### 3.5 Ablation study

We carried out an ablation study to investigate how the attention mechanism and Normalized Temperature-scaled Cross Entropy Loss (NT-Xent loss) function (Sohn 2016) affect the modeling ability of IGCN. Particularly, we developed three variants of IGCN: (1) we disabled the attention mechanism, and computed node embeddings as an average of node embeddings from each network; (2) we disabled the NT-Xent loss function for all omics networks (shown in Fig. 1); and (3) we used the proposed IGCN architecture to observe how disabling of different components affect the model performance. We conducted the experiments on TCGA-BRCA and ROSMAP datasets and reported the classification performance with macro F1 score measure in Table 4. The results show that both the attention mechanism module and the NT-Xent loss function are vital in node classification tasks, as the full IGCN setup achieved the best performance. Therefore, directly combining different types of omics data embeddings without accounting for the sample-level importance of various networks, provided by the attention module, may lead to incorrect predictions and degrade performance. Additionally, some edges in the graph may not accurately represent the true interactions or relationships between nodes. These inaccuracies can adversely affect classification tasks. The results demonstrate that the NT-Xent loss can alleviate these inaccuracies and boost the classification performance of IGCN.

**Table 4.** The average macro F1 scores of different variants of IGCN on TCGA-BRCA and ROSMAP datasets over ten runs.^a^

Components		Macro F1	
Attn. Mech.	$L_{NT-\mathrm{Xent}}$	TCGA-BRCA	ROSMAP
**✗**	✓	0.829±0.023	0.781±0.021
✓	×	0.831±0.018	0.811±0.035
✓	✓	**0.852 ± 0.017**	**0.823 ± 0.034**

a Attn. Mech.: Attention mechanism, $L_{NT-\mathrm{Xent}}$: Normalized Temperature-scaled Cross Entropy Loss.

## 4 Discussion

Due to the rapid advancements in omics technologies, along with major studies such as TCGA, ROSMAP, and ADNI, multi-omics datasets have become prevalent. Therefore, creating computational tools for the integrative analysis of various omics data types has been critically important in cancer molecular biology and precision medicine research. Toward this goal, to advance personalized medicine and uncover previously unknown characteristics through integrative analysis of multi-omics data, we present IGCN as a framework to integrate multi-omics datasets. To demonstrate the superiority of IGCN, we not only compare its performance with other multi-omics integrative tools but also compare it with recently introduced multi-modal graph representation learning methods, which have not been applied for multi-omics integration.

IGCN utilizes multi-GCN modules to extract node embeddings from each omics data. The personalized attention mechanism in IGCN is designed to integrate multi-omics data embeddings into a weighted form. This module assigns omic type attention values specific for each sample, allowing us to observe the unique characteristic patterns of each omics type within the label space. Our findings revealed that the importance of different embeddings from various omics data networks changes for each sample (Fig. 3). In line with previous research, where mRNA expression data has been consistently recognized as a key factor in identifying breast cancer subtypes, our analysis of the TCGA-BRCA dataset confirms that mRNA expression significantly contributed to the prediction of Basal-like, HER2-enriched, and Luminal A subtypes. This is expected, given that PAM50 subtypes were traditionally defined based on mRNA expression profiles. Notably, however, our findings also reveal that miRNA expression data was the most influential in predicting the Normal-like breast cancer subtype, which adds a new dimension to the understanding of breast cancer classification and highlights the potential of miRNA as a complementary datatype in subtype prediction.

Moreover, IGCN’s ability to assign attention coefficients to features within individual samples across various omics data types represents a significant advancement. This capability not only aids in identifying key biomarkers in the pathogenesis of diseases such as BRCA, GBM, and AD but also suggests specific genes as candidates for predicting and differentiating cancer subtypes and other biomedical outcomes at the molecular level (Tables 2 and 3). By effectively highlighting biomarkers across different cancer molecular subtypes and disease types, IGCN enhances our understanding of the underlying molecular mechanisms driving disease heterogeneity. This insight supports more accurate subtype classification, ultimately leading to more personalized and targeted therapeutic strategies.

One challenge encountered in our study was the limited availability of data across all modalities for some patients, which resulted in a small sample size. However, as more samples and omics layers become available, future studies could conduct a more robust and extensive analysis. Such enriched data would allow for a deeper exploration of the complex interactions between different biological layers, ultimately enhancing our ability to uncover meaningful insights and improve predictive accuracy. Another important aspect to consider in our study is the use of datasets derived from bulk tissue samples rather than single-cell data. While bulk tissue analysis offers valuable insights into overall gene expression patterns, it can obscure the cellular heterogeneity within the tissue. Single-cell omics datasets would provide a more detailed view of the diversity among individual cells, enabling a deeper understanding of the specific roles of different cell populations in pathophysiology.

## Supplementary Material

btaf313_Supplementary_Data

## Data Availability

TCGA-BRCA and ROSMAP samples with mRNA expression, DNA methylation, and miRNA expression data were collected from the TCGA project data portal (available at https://portal.gdc.cancer.gov) and ROSMAP cohort (available at https://dss.niagads.org/cohorts/religious-orders-study-memory-and-aging-project-rosmap/), respectively. We utilized the preprocessed data provided by (Wang *et al.* 2021) (available at https://github.com/txWang/MOGONET). For the GBM subtype classification task, we obtained mRNA and miRNA expression data from the Broad Institute’s Firehose portal (Deng *et al.* 2017) (available at http://firebrowse.org/). DNA methylation data was excluded due to limited sample availability. We also collected various omics types, including single nucleotide polymorphisms, lipidomics, and bileomics, from the ADNI dataset (Petersen *et al.* 2010) (https://adni.loni.usc.edu/) to classify AD, MCI, and CN status.

## Code availability

The source code and documentation is available at https://github.com/bozdaglab/IGCN and https://doi.org/10.5281/zenodo.15345835.
