# Supplementary material for: IGCN: integrative graph convolution networks for patient level insights and biomarker discovery in multi-omics integration
Source: Bioinformatics. 2025 Jun 4;41(6):btaf313. doi: 10.1093/bioinformatics/btaf313 (PMC12204196; doi:10.1093/bioinformatics/btaf313)
Supplement: btaf313_Supplementary_Data [file btaf313_supplementary_data.pdf]

IGCN: Integrative Graph Convolution Networks for patient level insights and biomarker discovery in multi-omics integration: supplemental document

Dataset	# Features before preprocessing	# Node features	# Samples (Groups)	# Edges
TCGA-BRCA	mRNA: 20,531<br>DNA methylation: 20,106<br>miRNA: 503	mRNA: 1,000<br>DNA methylation: 1,000<br>miRNA: 503	Basal-like: 131<br>HER2-enriched: 46<br>Luminal A: 436<br>Luminal B: 147<br>Normal-like: 115	mRNA: 2,629<br>DNA methylation: 2,629<br>miRNA: 2,631
TCGA-GBM	mRNA: 12,044<br>miRNA: 536	mRNA: 1,230<br>miRNA: 534	Proneural: 164<br>Neural: 97<br>Mesenchymal: 138<br>Classical: 120	mRNA: 1,559<br>miRNA: 1,559
ROSMAP	mRNA: 55,889<br>DNA methylation: 23,788<br>miRNA: 309	mRNA: 200<br>DNA methylation: 200<br>miRNA: 200	CN: 169<br>AD: 182	mRNA: 1,055<br>DNA methylation: 1,055<br>miRNA: 1,055
ADNI	SNPs: 2,126,516<br>Lipidomics: 781<br>Bileomics: 24	SNPs: 156<br>Lipidomics: 637<br>Bileomics: 24	CN: 214<br>MCI: 210<br>AD: 178	SNPs: 1,808<br>Lipidomics: 1,808<br>Bileomics: 1,808

Table S1. Description of the multi-omics datasets. CN: Cognitively Normal, MCI: Mild Cognitive Impairment, AD: Alzheimer's Disease.

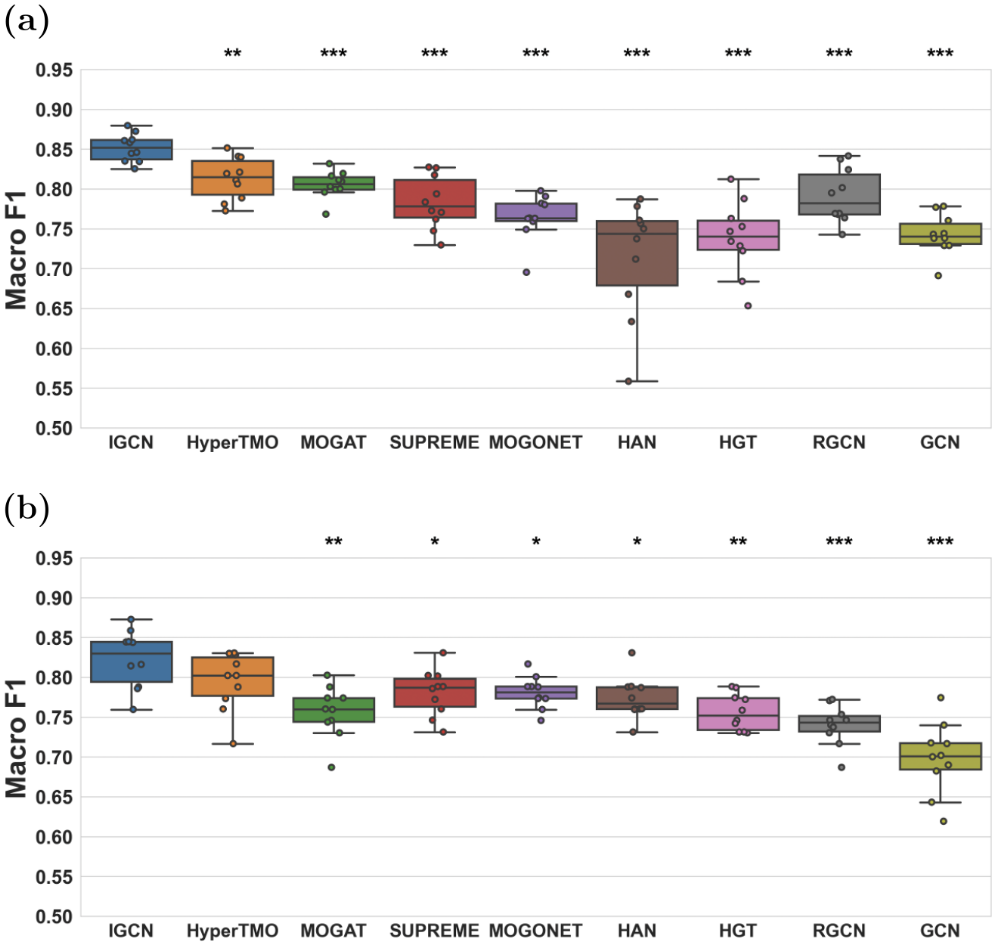

Fig. S1. The boxplots show the distribution of macro F1 scores of ten different runs on **(a)** TCGA-BRCA and **(b)** ROSMAP datasets for all methods. Wilcoxon rank-sum test p-values were computed between IGCN and other methods to compare the distribution of box plots. ***:p-value $<$ 0.001, **:p-value $<$ 0.01, *:p-value $<$ 0.05.

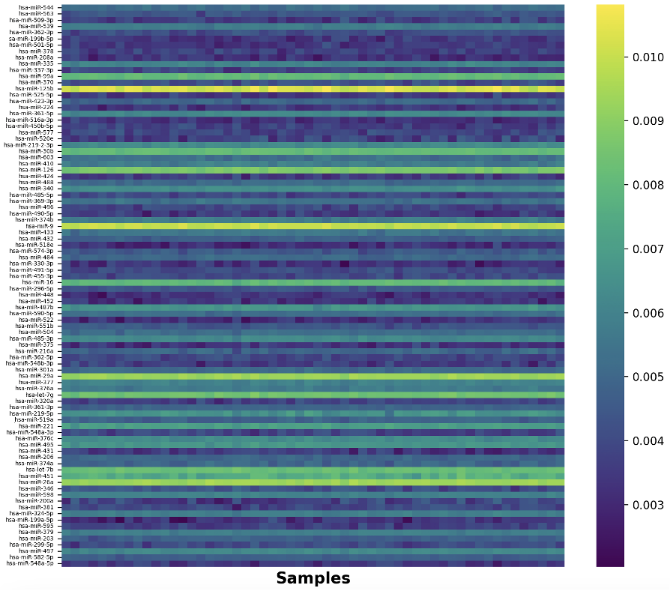

Fig. S2. The attention values for miRNA features. The distribution of attention values exhibits notable variations across the features.

(a)

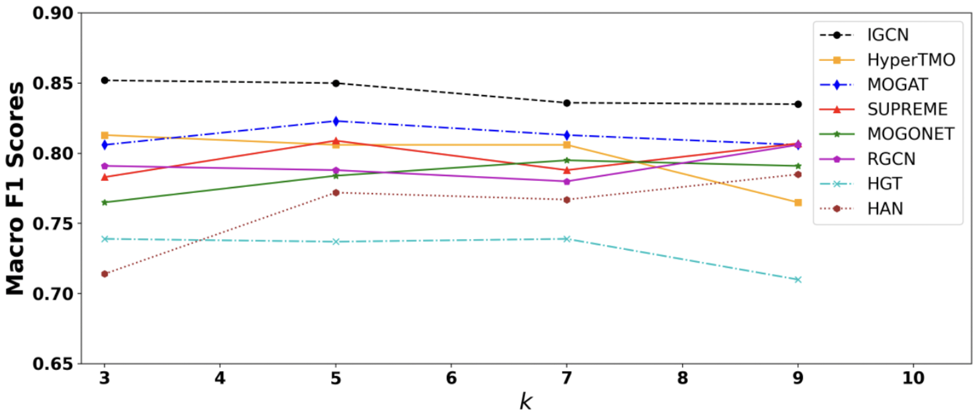

(b)

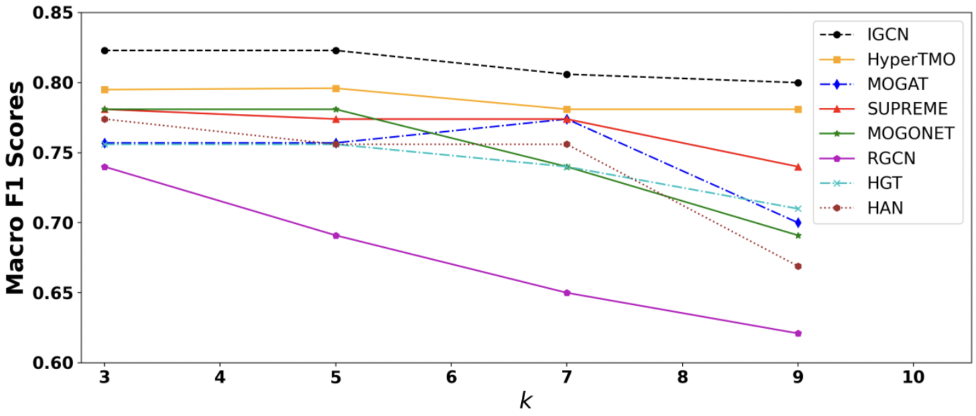

Fig. S3. The average macro F1 scores of IGCN, HyperTMO, MOGAT, SUPREME, MOGONET, RGCN, HGT, and HAN on **(a)** TCGA-BRCA and **(b)** ROSMAP datasets based on different k values, which represent the average node degree of the similarity network.

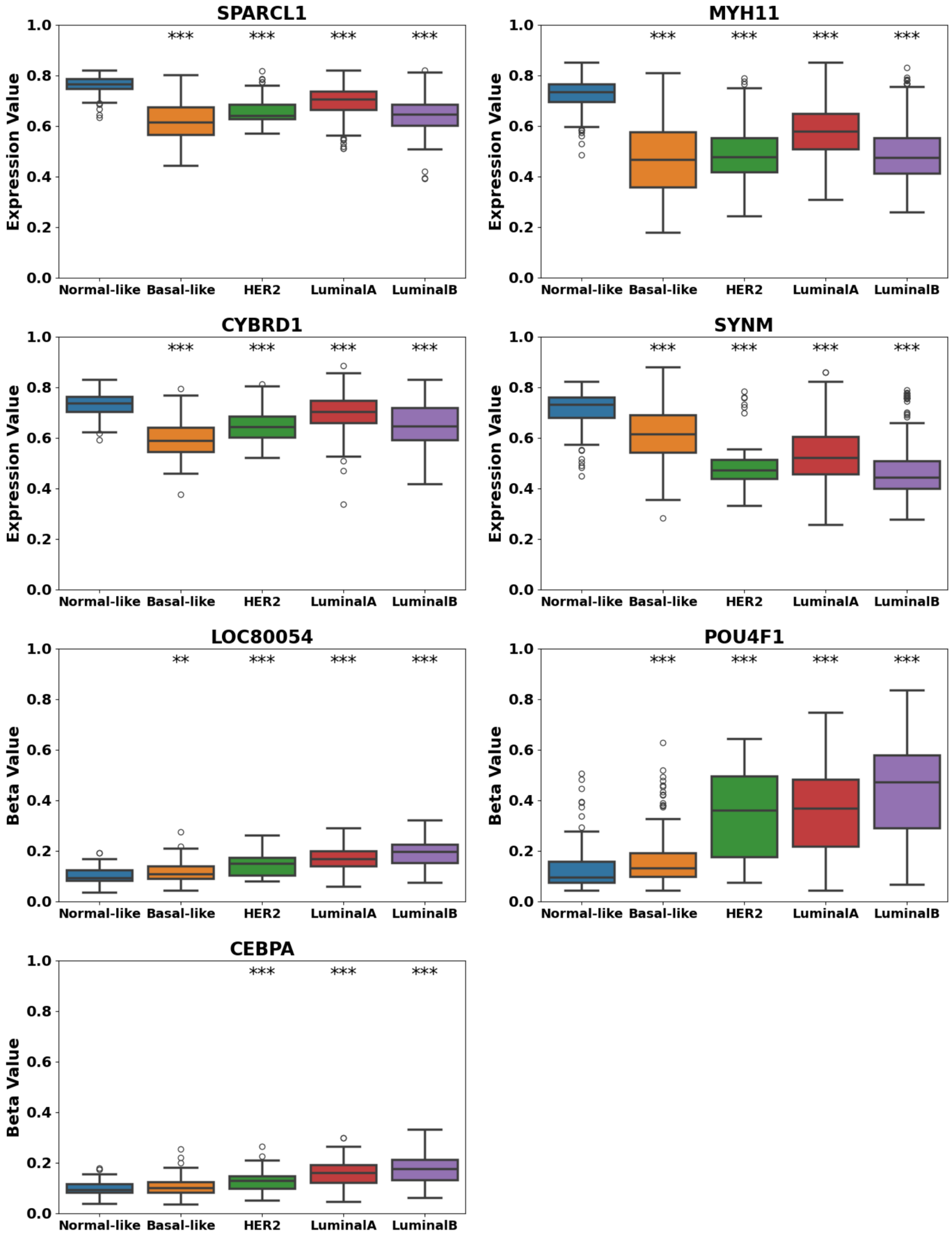

Fig. S4. Distribution of expression/DNA Methylation values of identified Normal-like specific biomarkers across BRCA PAM50 subtypes. Box plots illustrate the variability and significance of each biomarker in distinguishing molecular subtypes. ***:p-value < 0.001, **:p-value < 0.01, *:p-value < 0.05.

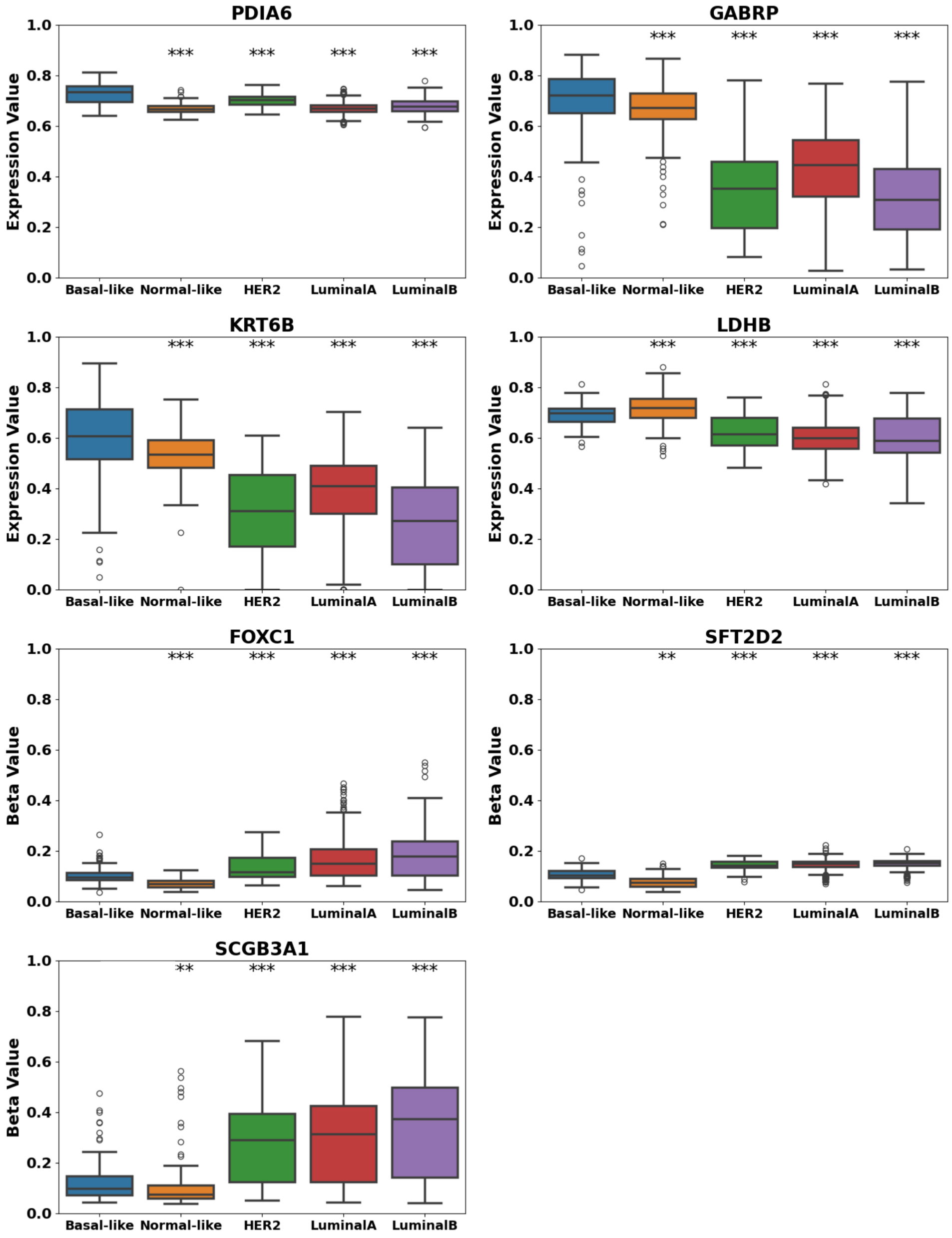

Fig. S5. Distribution of expression/DNA Methylation values of identified Basal-like specific biomarkers across BRCA PAM50 subtypes. Box plots illustrate the variability and significance of each biomarker in distinguishing molecular subtypes. ***:p-value < 0.001, **:p-value < 0.01, *:p-value < 0.05.

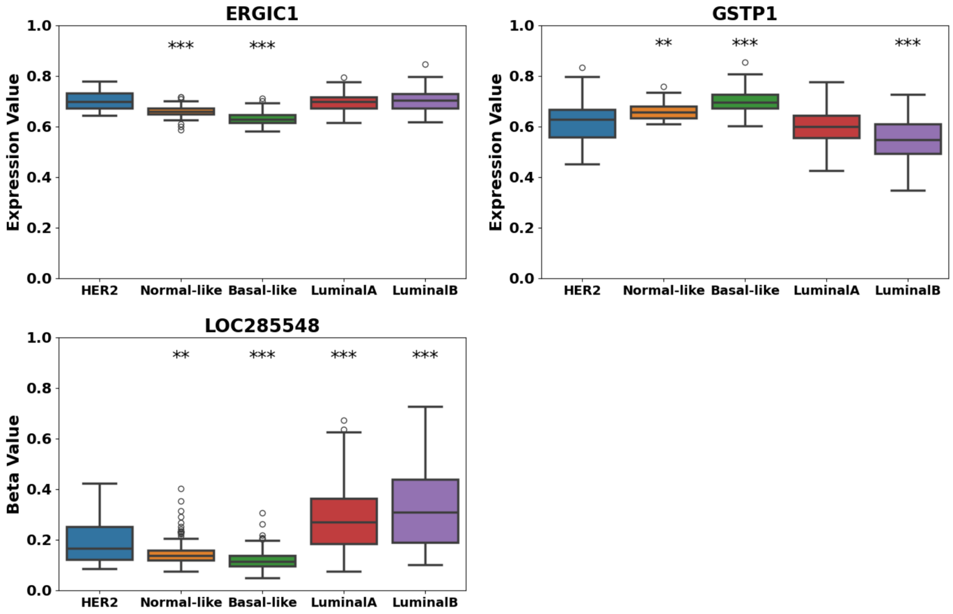

Fig. S6. Distribution of expression/DNA Methylation values of identified HER2 specific biomarkers across BRCA PAM50 subtypes. Box plots illustrate the variability and significance of each biomarker in distinguishing molecular subtypes. ***:p-value $<$ 0.001, **:p-value $<$ 0.01, *:p-value $<$ 0.05.

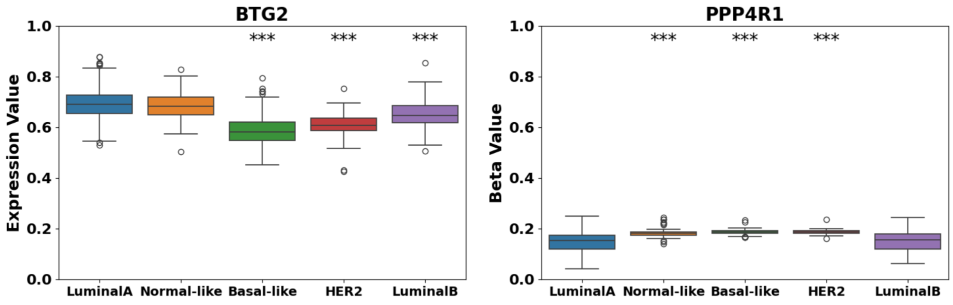

Fig. S7. Distribution of expression/DNA Methylation values of identified Luminal A specific biomarkers across BRCA PAM50 subtypes. Box plots illustrate the variability and significance of each biomarker in distinguishing molecular subtypes. ***:p-value $<$ 0.001, **:p-value $<$ 0.01, *:p-value $<$ 0.05.

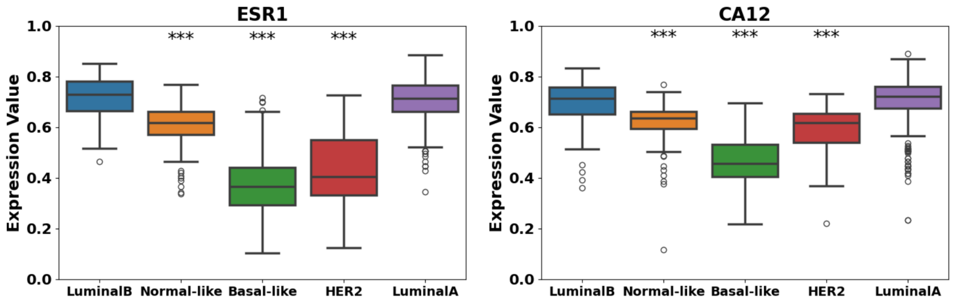

Fig. S8. Distribution of expression/DNA Methylation values of identified Luminal B specific biomarkers across BRCA PAM50 subtypes. Box plots illustrate the variability and significance of each biomarker in distinguishing molecular subtypes. ***:p-value < 0.001, **:p-value < 0.01, *:p-value < 0.05.
